# No effects of respiratory muscle endurance exercises on postprandial glucose excursions

**DOI:** 10.3389/fspor.2026.1831908

**Published:** 2026-05-28

**Authors:** Fernando G. Beltrami, Joel Mullarkey, Christina M. Spengler

**Affiliations:** 1 Exercise Physiology Lab, Department of Health Sciences and Technology, ETH Zurich, Zurich, Switzerland; 2 Zurich Center for Integrative Human Physiology (ZIHP), University of Zurich, Zurich, Switzerland

**Keywords:** management—healthcare, pre-diabetes (pre-DM), prevention, respiratory muscle, type II diabetes (T2D)

## Abstract

**Introduction:**

Whole-body exercise can attenuate postprandial glucose (PPG) excursions, one of the three main components in glucose control. Respiratory muscle endurance exercises (RMEE) based on resisted hyperpnea could offer an alternative or managing postprandial glucose (PPG) excursions in individuals unable or unwilling to perform whole-body exercise.

**Methods:**

We investigated whether PPG excursions during a 2-h oral glucose tolerance test are attenuated with 5 min (RMEE-5) or 15 min (RMEE-15) of respiratory muscle endurance exercises in 18 younger (age 24 ± 4 years) and 10 older (age 54 ± 7 years) healthy adults. On three separate visits, participants (8-h fasted) ingested 75 g of glucose and, 14min later, performed either RMEE-5, RMEE-15, or sat quietly (control). Blood glucose concentration was monitored periodically for a 2-h period.

**Results:**

Neither intervention differed from control in terms of glucose area under the curve (*p* > 0.53), peak glucose (*p* > 0.09), time to peak glucose (*p* > 0.21), or glucose level at 2 h (*p* > 0.60).

**Discussion:**

Based on the apparent lack of effects regardless of age group and exercise duration, bouts of increased respiratory muscle work do not seem to be effective for the management of PPG.

## Introduction

Insufficient blood glucose control leads to hyperglycemia, increasing the risk for a variety of macro- and microvascular diseases (1–3). Blood glucose behavior is often described in terms of the glucose triad, which consists of increased fasting blood glucose levels, glycated hemoglobin (Hb_A1c_) and postprandial glucose (PPG) excursion (4). While the former two have long received substantial attention in the literature, there is growing appreciation of the positive effects of lowering PPG excursions in the management of diabetes, including a lowering of Hb_A1c_ and reductions of cardiovascular risk (5, 6).

Physical exercise can reduce blood glucose values by increasing skeletal muscle glucose uptake independently of insulin release (7, 8). The main mechanisms involved in this process include a greater delivery of glucose to skeletal muscle, an increase in the permeability of glucose over the surface membrane in skeletal muscle and an increase in the intracellular substrate flux due to increased intracellular metabolism (9). Whole-body physical exercise can blunt PPG excursions in both healthy and diabetic individuals, an effect which is additive to glucose lowering medication (10, 11).

Despite the potential benefits of physical exercise in the management of PPG excursions, a large fraction of the population remains unwilling or is unable to exercise following meals and therefore could profit from alternative modalities, especially if these could be performed while sitting. Early evidence suggests that increasing inspiratory muscle load affects blood glucose levels (12), offering the possibility that respiratory exercises might be a useful tool in the management of hyperglycemia. Respiratory muscle endurance exercise (RMEE) imposes a cardiovascular load (13) and energy expenditure (14) of 1–2 metabolic equivalents net of resting energy expenditure, similar to light walking. Therefore, RMEE could provide sufficient muscular work to reduce PPG while being more conveniently performed, particularly if an effect can be found with low dosages, which could facilitate adherence. This possibility, however, has not yet been tested.

## Research design and methods

### Participants

Sample size calculations were performed on G*Power (15), based on an alpha of 0.05 and statistical power of 80%, using an effect size of 0.75 for a two-tailed pairwise comparison, in line with the effect sizes reported after aerobic interventions of similar duration in PPG variables (16), resulting in a sample size of 16 individuals. Eighteen younger (<45 years) took part in the first phase of the study, and ten older (>45 years) adults participated in a follow-up, second phase of the study, which was interrupted prior to the originally planned 18 participants (see results). Characteristics of the participants, including lung function data, are presented in Table 1. Participants gave written informed consent, and the study was approved by the local ethics committee (KEK 2020-01778).

**Table 1:** Participants' characteristics.

Variable	Younger (*n* = 18)	Older (*n* = 10)
Male: Female	9 : 9	5 : 5
Age (years)	23.5 ± 3.8	54.1 ± 6.5
Height (cm)	175 ± 9	176 ± 10
Body mass (kg)	70.6 ± 13.5	80.5 ± 13.0
Body fat %	25 ± 7	33 ± 7
Resting heart rate (bpm)	75 ± 11	69 ± 11
FVC (l)	5.3 ± 1.1	4.5 ± 1.2
FVC % pred.	115 ± 10	105 ± 11
FEV_1_/FVC	0.82 ± 0.07	0.79 ± 0.05
MVV (l)	163 ± 40	148 ± 51
MVV % pred.	121 ± 15	122 ± 27
MIP (cm H_2_O)	116 ± 31	125 ± 40
MIP % pred.	118 ± 20	154 ± 40
MEP (cm H_2_O)	148 ± 47	199 ± 61
MEP %pred.	115 ± 19	173 ± 49

Data as mean ± SD. % pred., percentage of predicted values; FVC, forced vital capacity; FEV_1_, forced expiratory volume during the first second; MVV, maximal voluntary ventilation; MIP, maximal inspiratory pressure; MEP, maximal expiratory pressure.

### Study design

This study was a randomized, cross-over trial. Participants completed four visits each. Visit 1 consisted of anthropometric measurements including body densitometry for determination of percentage body fat (Dual Energy x-Ray Absorptiometry, GE Healthcare, Madison, WI, USA), assessments of lung function and respiratory muscle strength tests as well as familiarization with the protocol. During visits 2–4 (performed within 2 weeks, always at the same time of day) blood glucose and heart rate were measured (every 5 min during the first hour and every 10 min for the second hour) in response to an oral glucose tolerance test (OGTT). Fourteen minutes after glucose ingestion participants performed either 5 min (RMEE-5) or 15 min of RMEE (RMEE-15), or remained sit (control), in randomized and balanced order. A delay of 14 min from glucose ingestion was chosen to optimize the balance between the time taken for the ingested glucose to appear in circulation and the estimated time of peak blood glucose given the high glycemic index of the solution ingested, namely from 30 min of glucose ingestion onwards (17, 18). Randomization was performed by first producing a list of with all possible test orders in balanced manner, and then assigning each participant to one of the possible orders using the random number generation function of Microsoft Excel (Microsoft, Redmond, US). Participants were instructed to arrive in the laboratory after at least 8 h of fasting, to refrain from alcohol and physical exercise on the day before a test and to keep their regular diets.

### Lung function and respiratory muscle strength tests

Vital capacity (VC), forced vital capacity (FVC) and maximal voluntary ventilation (MVV) were determined using a calibrated metabolic cart (Jaeger Oxycon Pro, Viasys Healthcare GmbH, Hoechberg, Germany), conforming to the current guidelines (19). Maximal inspiratory and expiratory pressures were measured at residual volume using a handheld device (MD Diagnostics Ltd., Maidstone, England). At least three maneuvers were performed, with the last one not representing the highest value and the two highest values varying by less than 5%.

### Respiratory exercises

RMEE consisted of either 5 min (RMEE-5) or 15 min (RMEE-15) of normocapnic hyperpnea using the SpiroTiger device (SpiroTiger®, Idiag, Fehraltorf, Switzerland), which has an adjustable bag that allows partial rebreathing of the expired air, to ensure normocapnia. The participants were breathing with a tidal volume of 60% of their forced vital capacity and the breathing frequency adjusted to reach their personal ventilatory target (younger adults, 60% (RMEE-15) or 75% (RMEE-5) of their maximal voluntary ventilation (MVV); older adults, 50% (RMEE-15) or 65% (RMEE-5) of MVV). The device was connected to the ergospirometer, and participants were shown their tidal volume for each breath on a screen. A metronome was used to guide breathing frequency. The participants were verbally encouraged throughout the protocol to achieve their target ventilation as closely as possible. At the end of the task, they were asked to indicate their perception of breathlessness and respiratory exertion on separate visual analog scales (20-cm wide), with values later transformed into a 0–10-point range. Both concepts were explained to the participants before the start of the tests (20).

### Oral glucose tolerance test

Before and during the OGTTs, 20-µL of arterialized blood was drawn from an ear lobe at standardized intervals and analyzed for glucose concentration (BIOSEN C_line Sport ®, EKFdiagnostic, Barleben, Germany). Samples were drawn 5 min before and immediately after the start of drinking the glucose solution (75 g of glucose mixed in 250 mL of water; ingested within 3 min); thereafter samples were collected every 5 min for the 1st hour and every 10 min for the 2nd hour. Baseline values were calculated as the average of the first two samples. Peak glucose was defined as the maximal value measured during the protocol and time-to-peak was defined from start of ingestion to the minute in which the peak occurred. The area under curve (AUC) was defined as the area under the glucose curve above baseline, during the 2 h after start of glucose ingestion, using the trapezoidal rule.

### Statistical analysis

Area under the curve, glucose peak and time-to-peak were analyzed with one-way repeated measures ANOVA, with Dunnet's *post hoc* when required to compare the RMEE-5 or RMEE-15 interventions against the control day. When required, data from the younger and older adults was compared using independent *t*-tests (2-tailed). Values are expressed as means ± SD. Statistical significance was set at *p* < 0.05. All analyzes were performed using Prism 10.0 (Graphpad, La Jolla, CA). Effect sizes are reported as partial eta squared for the repeated measures ANOVA.

## Results

### Blood glucose

Resting baseline blood glucose values were not different between days in younger (*p* = 0.863, $\eta_{P}^{2}=0.01$) and older (*p* = 0.575, $\eta_{P}^{2}=0.05$) participants.

**Figure 1:**
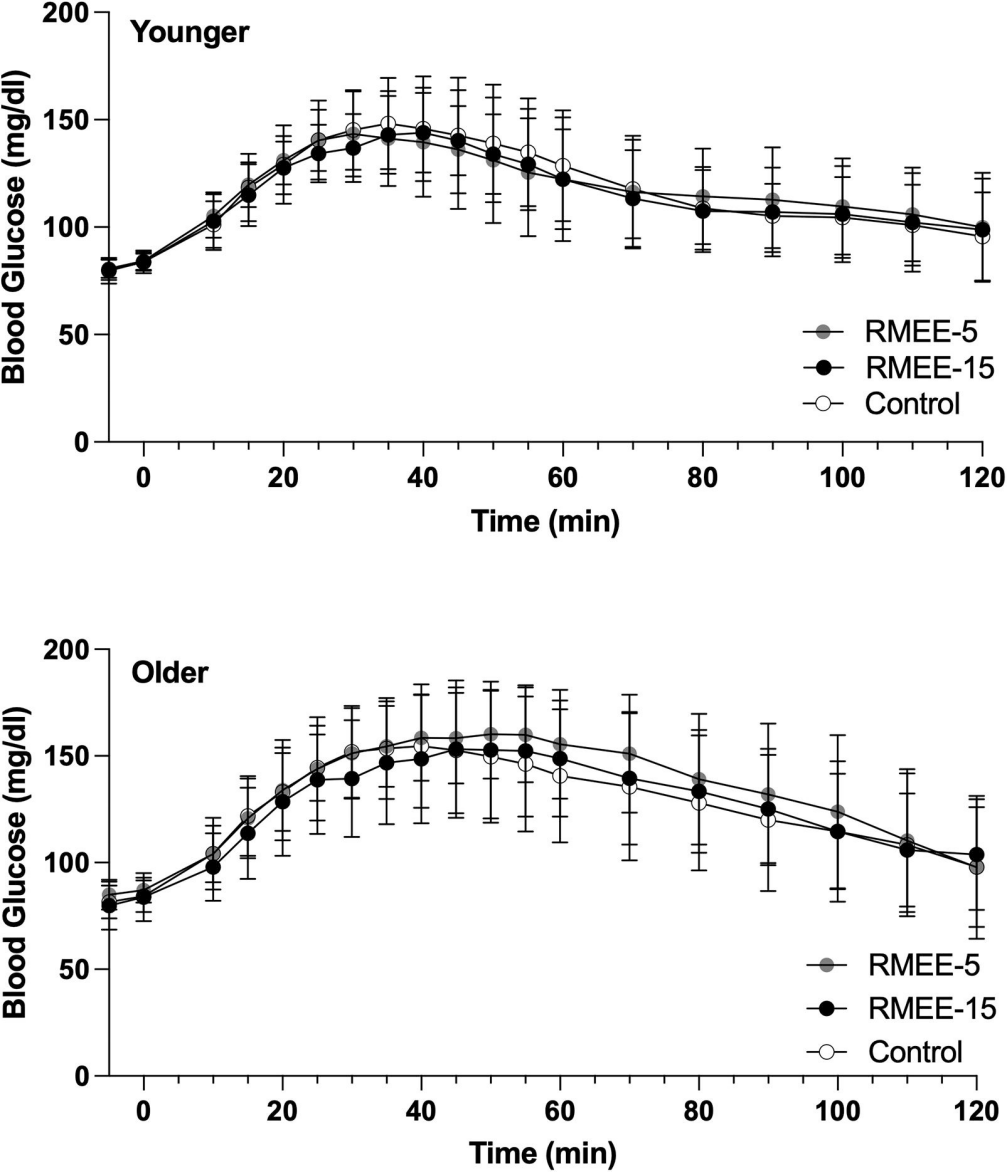
Blood glucose excursions for younger (top) and older (bottom) adults during a 2-h oral glucose tolerance test. 14 min after glucose ingestion, participants either performed 5 min of normocapnic hyperpnea (RMEE-5) or 15 min of normocapnic hyperpnea (RMEE-15) or they sat quietly (control).

**Figure 2:**
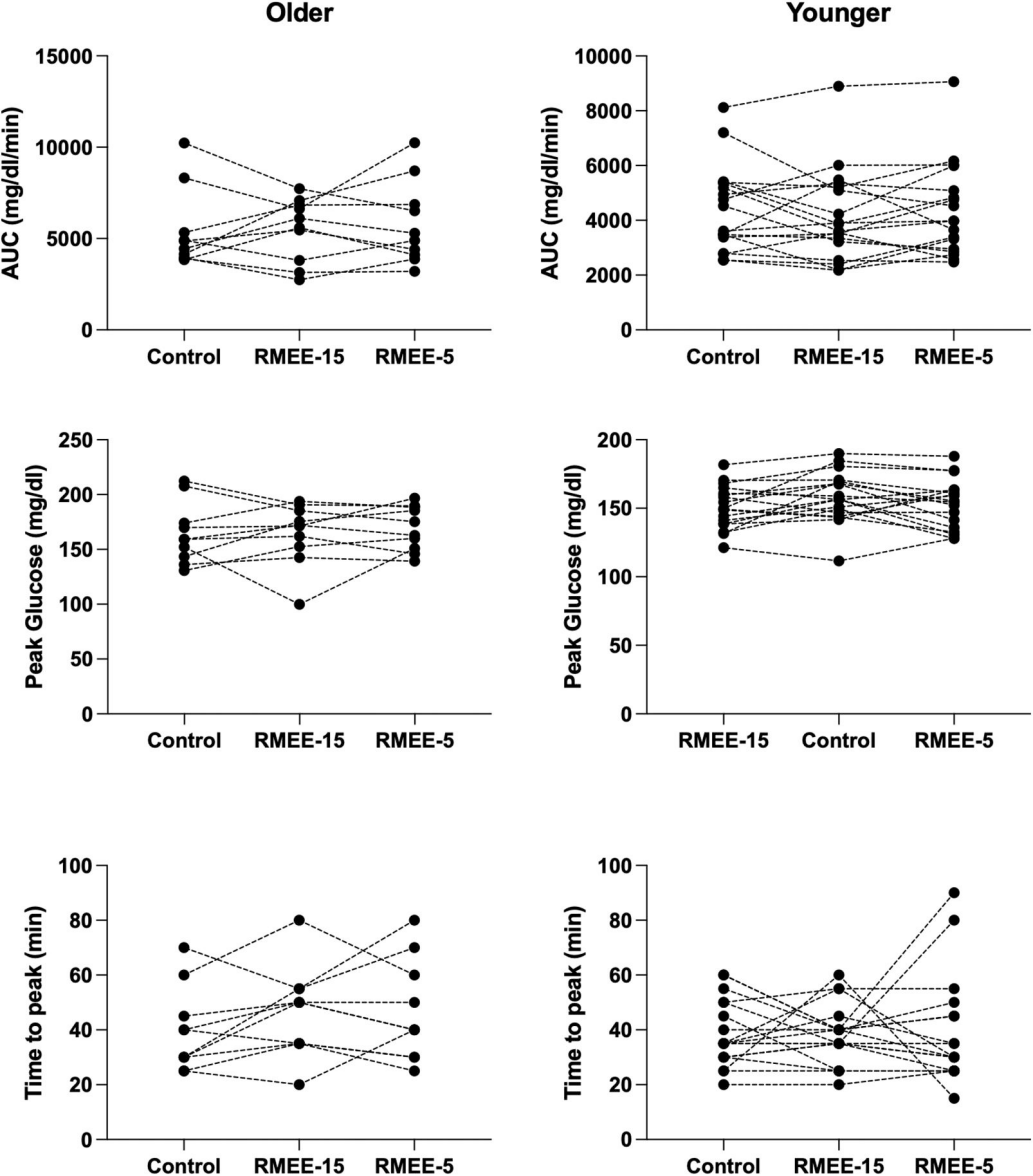
Individual value for the older (left) and younger (right) participants. Top, Glucose area under the curve (AUC) during the oral glucose tolerance test (OGTT); Middle, peak glucose concentration during the OGTT; bottom, time to peak glucose during the OGTT. Control, control intervention (quiet sitting); RMEE-15, 15 min of resisted hyperpnea; RMEE-5, 5 min of resisted hyperpnea.

The PPG excursion curves with the different interventions are presented in Figure 1 and Table 2. Individual values for glucose AUC, peak glucose and time to peak are presented in Figure 2. Neither RMEE-5 nor RMEE-15 changed glucose AUC (younger, *p* = 0.537, $\eta_{P}^{2}=0.03$; older, *p* = 0.742, $\eta_{P}^{2}=0.03$), peak glucose (younger, *p* = 0.093, $\eta_{P}^{2}=0.13$; older *p* = 0.728, $\eta_{P}^{2}=0.03$), time to peak glucose (younger *p* = 0.960, $\eta_{P}^{2}=0.00$; older *p* = 0.217, $\eta_{P}^{2}=0.16$), or glucose values at 2 h (younger *p* = 0.609, $\eta_{P}^{2}=0.03$; older *p* = 0.697, $\eta_{P}^{2}=0.03$).

**Table 2:** Blood glucose responses to different interventions during an oral glucose tolerance test (OGTT).

Variable	Younger (n = 18)			ANOVA	Older (n = 10)			ANOVA
	RMEE-5	RMEE-15	Control		RMEE-5	RMEE-15	Control	
Resting blood glucose (mg/dL)	84 ± 4	84 ± 4	84 ± 5	F_(1.6, 14.6)_ = 0.5	87 ± 6	84 ± 11	84 ± 7	F_(1.9, 32.6)_ = 0.1
Time to peak glucose (min)	39 ± 20	38 ± 11	39 ± 12	F_(1.9, 17.4)_ = 1.7	47 ± 18	47 ± 16	40 ± 15	F_(1.4, 24.1)_ = 0.0
Peak blood glucose (mg/dL)	153 ± 18	151 ± 16	158 ± 18	F_(1.9, 16.8)_ = 0.3	170 ± 20	165 ± 28	165 ± 28	F_(2.0, 33.8)_ = 2.5
2 h blood glucose (mg/dL)	100 ± 25	99 ± 24	96 ± 20	F_(1.7, 15.6)_ = 0.3	98 ± 34	104 ± 26	98 ± 28	F_(1.7, 28.3)_ = 0.4
2 h AUC (mg.min/dL)	4,356 ± 1,682	4,145 ± 1,669	4,390 ± 1,571	F_(1.8, 16.2)_ = 0.3	5,812 ± 2,266	5,513 ± 1,729	5,393 ± 2,149	F_(1.9, 32.2)_ = 0.6

Data are mean ± SD. ANOVA column shows the F statistic for the main treatment effect. AUC, area under the curve calculated via the trapezoid method. None of the parameters showed a difference compared with control.

Pooling the data of the younger and older participants together showed similar results: there was no detectable effect of intervention for glucose AUC (*p* = 0.622, $\eta_{P}^{2}=0.02$), peak glucose (*p* = 0.363, $\eta_{P}^{2}=0.04$), time to peak glucose (*p* = 0.626, $\eta_{P}^{2}=0.02$) or glucose at 2 h (*p* = 0.587, $\eta_{P}^{2}=0.02$). Splitting the cohort of younger adults into males and females did not show any interaction of sex and intervention on the evaluated parameters (not shown). This procedure was not carried out with the older adults due to lack of statistical power.

### Respiratory muscle endurance exercise

Table 3 shows the physiological and perceptual data from the RMEE-5 and RMEE-15 interventions. Heart rate increased by 25–30 bpm during the two hyperpnea trials compared with quiet sitting (control). The pulmonary ventilation achieved during the exercise was close to the targets, indicating that most participants either successfully reached the desired level of respiratory muscle work or were working close to their capacity. In both the RMEE-5 and RMEE-15 trials, the level of breathlessness reported at the end of the trial was low, with no difference between conditions in younger (*p* = 0.139) and older (*p* = 0.286) adults. Levels of respiratory exertion were not different between RMEE-5 and RMEE-15 in younger adults (*p* = 0.364) but higher levels of respiratory exertion in older adults following RMEE-15 compared with HYP5 (*p* = 0.042).

**Table 3:** Physiological and perceptual responses to respiratory muscle exercise.

Variable	Younger (*n* = 18)		Older (*n* = 10)	
	RMEE-5	RMEE-15	RMEE-5	RMEE-15
V˙_E_ (l/min)	104 ± 28	91 ± 23	75 ± 25	68 ± 22
V˙_E_ (%MVV)	56 ± 3	46 ± 4	64 ± 6	51 ± 4
V_T_ (l)	2.9 ± 0.5	3.0 ± 0.6	1.9 ± 0.7	1.9 ± 0.6
f_B_ (breaths/min)	34 ± 6	30 ± 4	41 ± 7	36 ± 5
HR (beats/min)	115 ± 19	104 ± 17	94 ± 13	93 ± 15
Breathlessness (points)	2.5 ± 2.4	1.5 ± 2.0	1.8 ± 2.3	2.8 ± 2.6
Respiratory exertion (points)	6.8 ± 2.4	6.3 ± 2.0	4.5 ± 2.3	5.9 ± 2.9

Data are mean ± SD. V̇_E_, minute ventilation; %MVV, percentage of maximal voluntary ventilation, V_T_, tidal volume, f_B_, breathing frequency; HR, heart rate.

## Discussion

This study investigated whether two different RMEE protocols could attenuate post-prandial glucose excursions in younger and older healthy adults. Contrary to our hypothesis, neither 5 nor 15 min of RMEE provided meaningful changes in PPG excursions. This was consistent across all metrics evaluated, including peak glucose, time-to-peak, area under the curve and blood glucose levels after 2 h. Finally, no effect of sex was noted for the any of the variables tested.

There is conflicting evidence regarding the potential for inspiratory muscle training, which can be seen as strengthening exercises for the respiratory muscles for assisting in glucose management. One acute study showed that strong (i.e., 60% of maximal inspiratory pressure) contractions of the diaphragm had acute effects on blood glucose in six diabetic patients (12), though the use of continuous glucose monitoring might have made results dependent on changes in blood flow to the arm during intense breathing maneuvers, as values quickly returned to controls levels immediately after exercise cessation. A subsequent study by the same group found no differences in glucose control compared to a placebo intervention (2% MIP) when inspiratory muscle exercises (60% MIP) were performed over three days (21). A randomized study investigating daily inspiratory muscle training for 12 weeks in diabetic patients could not find evidence of improved glucose control (both Hb_A1c_ and fasting glucose) (22). However, training was performed at only 30% MIP and no improvement in MIP occurred, even though respiratory muscle endurance improved. Therefore, it could be argued that in the latter study the training load used was sub-optimal.

In the current study, a different approach was used, based on endurance exercise. Although caloric expenditure was not directly measured, the O_2_ cost oy hyperpnea can be calculated. Based on available equations (14), the caloric cost of unresisted hyperpnea in the young cohort of the current study would be 5.7 ± 2.6 kcal for RMEE-5 and 13.5 ± 6.2 kcal for RMEE-15. This is the equivalent of 1.4 ± 0.6 g and 3.4 ± 1.5 g of glucose respectively, or 1.9 ± 0.9% and 4.5% ± 2.0% of the ingested 75 g of glucose. These figures are an underrepresentation of the true cost of hyperpnea, since breathing was performed against a resistance, increasing the work performed by the respiratory muscles. While it could be argued that the overall caloric expenditure was simply too low to elicit any effect on PPG, it has been shown that a single minute of stair climbing, eliciting a caloric expenditure of 7 ± 2 Kcal, was sufficient to decrease peak glucose concentration and 60-min glucose AUC in pre-diabetic patients, although longer stair climbing trials elicited larger effects (23). Directly comparing the latter study with the present trial, stair climbing elicited an effect size for glucose AUC (Cohen D_z_) of 0.28 for 1-min and 0.44 for 3-min of exercise, whereas in the current study the effect sizes with the pooled participants (*n* = 28) was 0.08 for both RMEE-5 and RMEE-15, with the differences for RMEE-15 being in the undesired direction (i.e., a higher AUC for RMEE-15 compared with control). Therefore, while our study was powered to detect larger differences, we do not believe that the effect sizes observed are indicative of meaningful effects. Indeed, the trial with the older participants was interrupted before all pre-defined participants were recruited once the lack of effects was determined at mid-point of the data collection.

The present protocols were chosen based on the effectiveness of RMEE protocols to elicit muscle fatigue and training responses (24, 25). These studies used 30-min sessions of RMEE, whereas we limited duration to 5 and 15 min to test whether smaller doses of exercise would be enough to trigger an effect in PPG, as seen with other aerobic activities. Several participants failed to reach the desired level of ventilation, implying maximal or near-maximal intensity. Therefore, the stimulus of RMEE could only be increased by increasing the duration of the sessions or the pressure generated each breath. Increasing the duration of the trials beyond 15 min, however, would also decrease the chances of high adherence, and therefore decrease the attractiveness of the intervention even if a positive effect had been found. As no effect of exercise on blood glucose was noted in the period where the RMEE was being performed, it is unlikely that extending the duration of the task would have yielded different outcomes.

As only glucose levels were measured, no mechanistic explanation for the lack of effects can be conclusively offered. It could be speculated that blood flow and therefore glucose delivery to tissues where glucose removal normally takes place could have been attenuated during RMEE: fatiguing contractions of the respiratory muscles increase sympathetic activity and eventually trigger the respiratory metaboreflex, both of which can reduce perfusion to resting muscles (26, 27). As skeletal muscles play a crucial role in removing blood glucose (28), decreased perfusion could in turn decrease the removal of blood glucose. The effects of RMEE on glucose dynamics, however, requires further investigation.

Whereas the current study was performed in healthy younger and older individuals, it could be asked whether these findings would be applicable to pre-diabetic or diabetic patients. The effects of exercise on PPG variables seem to be similar and direction and magnitude between healthy and diabetic patients, although no direct comparisons are available in literature (29). Nonetheless, there are known molecular pathways that could account for a lower effect of exercise on diabetic patients, including mitochondrial dysfunction and the use of medication that inhibits mitochondrial activity (30). Therefore, while the exclusive use of healthy individuals constitutes a limitation of our study, a larger effect of the tested intervention should have been expected. As the present results were clearly negative, there is very little chance that large effect sizes would be detected in pre- or diabetic individuals.

In summary, neither 5 min nor 15 min of RMEE reduced blood glucose levels during an OGTT performed by younger and older healthy individuals. Therefore, the activities performed were unable to shift the balance between glucose appearance and removal from circulation. While it is unclear whether glucose utilization is too small and/or whether changes in glucose clearance and production mitigate any positive effects, at this point respiratory interventions based on increased respiratory work cannot be advocated for the control of postprandial glucose.

## Data Availability

The original contributions presented in the study are included in the article/Supplementary Material, further inquiries can be directed to the corresponding author.
